# Multivalent Adhesion Molecule 7 Clusters Act as Signaling Platform for Host Cellular GTPase Activation and Facilitate Epithelial Barrier Dysfunction

**DOI:** 10.1371/journal.ppat.1004421

**Published:** 2014-09-25

**Authors:** Jenson Lim, Daniel H. Stones, Catherine Alice Hawley, Charlie Anne Watson, Anne Marie Krachler

**Affiliations:** Institute of Microbiology and Infection, School of Biosciences, University of Birmingham, Edgbaston, Birmingham, United Kingdom; Collège de France, France

## Abstract

*Vibrio parahaemolyticus* is an emerging bacterial pathogen which colonizes the gastrointestinal tract and can cause severe enteritis and bacteraemia. During infection, *V. parahaemolyticus* primarily attaches to the small intestine, where it causes extensive tissue damage and compromises epithelial barrier integrity. We have previously described that Multivalent Adhesion Molecule (MAM) 7 contributes to initial attachment of *V. parahaemolyticus* to epithelial cells. Here we show that the bacterial adhesin, through multivalent interactions between surface-induced adhesin clusters and phosphatidic acid lipids in the host cell membrane, induces activation of the small GTPase RhoA and actin rearrangements in host cells. In infection studies with *V. parahaemolyticus* we further demonstrate that adhesin-triggered activation of the ROCK/LIMK signaling axis is sufficient to redistribute tight junction proteins, leading to a loss of epithelial barrier function. Taken together, these findings show an unprecedented mechanism by which an adhesin acts as assembly platform for a host cellular signaling pathway, which ultimately facilitates breaching of the epithelial barrier by a bacterial pathogen.

## Author Summary

*Vibrio parahaemolyticus* is a bacterial pathogen which occurs in marine and estuarine environments. It is a main cause of gastrointestinal illness following the consumption of raw or undercooked seafood. In immunocompromised people, the bacteria can sometimes enter the bloodstream and cause septicemia, a serious and often fatal condition. *V. parahaemolyticus* attaches to host tissues using adhesive proteins. Multivalent Adhesion Molecule (MAM) 7 is an adhesin which helps the bacteria to hold onto the host cells early on during infection. It does so by binding two different molecules on the host, a protein (fibronectin) and phospholipids called phosphatidic acids. We show that MAM7 does not only play a role in sticking to host cells. By forming adhesin clusters on the host surface and binding to host lipids, it triggers signaling processes in the host. These include activation of RhoA, an important mediator of cytoskeletal dynamics. By doing so, MAM7 perturbs proteins at cellular junctions, which normally maintain the cells in the gut as a tightly sealed layer protective of environmental influences. When bacteria use MAM7 to attach to the intestine, the seals between cells break, permitting bacteria to cross the barrier and cause infection of underlying tissues.

## Introduction

*Vibrio parahaemolyticus* is an emerging food- and waterborne bacterial pathogen. It is predominantly associated with gastroenteritis but occasionally manifests as wound infection [1]–[3]. Although infections are self-limiting in immunocompetent patients, in rare cases, usually occurring in patients with an underlying primary disease, *V. parahaemolyticus* can rapidly disseminate into the blood stream and cause septicemia, a life-threatening condition [4], [5]. During gastrointestinal disease, the pathogen predominantly colonizes the distal small intestine, where it causes fluid accumulation, extensive tissue damage, a reduction in epithelial barrier function and inflammation [6]. *V. parahaemolyticus* virulence has so far mainly been attributed to secreted haemolysins (TDH and TRH) as well as a range of effector proteins secreted into the host cell cytoplasm via two type III secretion systems (T3SS1 and T3SS2) [7], [8]. *V. parahaemolyticus*-mediated cellular toxicity has been attributed to the effects of T3SS1 secreted proteins: VopS, VopQ and VPA0450 contribute to cell rounding, disruption of autophagic turnover and cell lysis, respectively [9]–[11]. T3SS2 has been implicated in intestinal colonization, cellular invasion and enterotoxicity [6], [12]. However, the increase in epithelial permeability seen during infection *in vivo* as well as in tissue culture models of infection has not been attributed to any particular virulence factor [6], [13]. Recently, we have shown that Multivalent Adhesion Molecule (MAM) 7, a constitutively expressed surface protein, contributes to pathogen attachment to host cells during the early stages of infection [14]. *V. parahaemolyticus* MAM7 recognizes two host surface receptors: it binds host membrane phosphatidic acid (PA) lipids with high affinity and uses the extracellular matrix protein fibronectin as a co-receptor. MAM7 contains seven mammalian cell entry (mce) domains and each individual domain is capable of binding PA, while a stretch of at least five repeats is required to interact with fibronectin. While PA binding is essential for attachment, binding to fibronectin is dispensable for the interaction but increases the on-rate of binding [15].

Both PA and fibronectin are important signaling molecules in their own right and are implicated in key cellular pathways. PAs make up an average 1–4% of a cell's total phospholipid content [16] and are important as precursors for the biogenesis of other phospholipids, in determining membrane curvature and as signaling molecules [17]–[19]. Several PA-binding proteins are known, including Raf-1, mTOR and SHP-1 [20]–[22]. As such, PAs are involved in the regulation of a diverse set of cellular functions, ranging from metabolism and trafficking to proliferation. Thus far, studies on PAs have focused on pathways involving PA localized in the inner leaflet of the plasma membrane and cellular organelles, such as the ER. Although PA can also be found in the outer leaflet of the plasma membrane, it is not characterized how this pool is generated or how it is linked to cellular functions [23], [24]. It has also been reported that PA generation in cells is localized to specific regions within the membrane, but the consequences of this compartmentalization are not well understood [25].

In this study, we found that the clustering of MAM7 molecules on the bacterial surface and subsequent binding of these clusters to phosphatidic acid lipids in the host membrane, causes downstream activation of the small GTPase RhoA. RhoA activation drives actin rearrangements which ultimately lead to redistribution of tight junction proteins and a disruption of epithelial integrity. This breach in the epithelial barrier allows *V. parahaemolyticus* to translocate across polarized epithelial layers. Thus, we report for the first time that a bacterial adhesin, through direct interactions with host lipid receptors, induces cellular signaling pathways facilitating epithelial barrier breaching by a bacterial pathogen.

## Results

### Local clustering of the adhesin MAM7 causes sustained actin rearrangements in host cells

**Figure 1:**
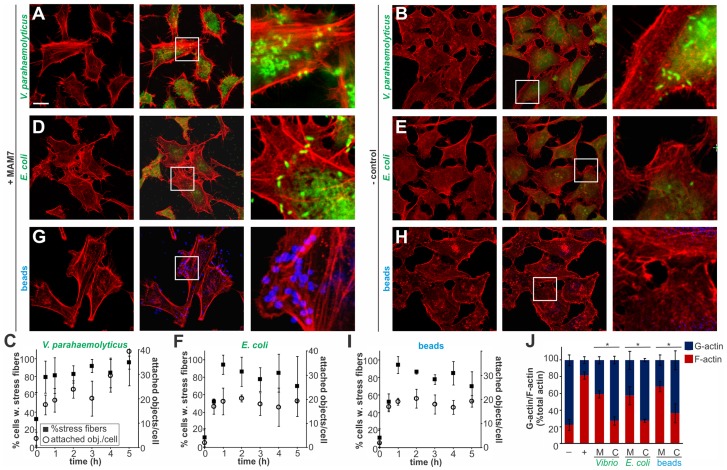
Clustering of MAM7 adhesin causes sustained actin rearrangements in host cells. Attachment of *V. parahaemolyticus* CAB4 or *E. coli* BL21-MAM7 (A, D, bacteria expressing MAM7 in green) or of polymer beads coupled to GST-MAM7 (G, beads in blue) to Hela cells caused sustained stress fiber formation (F-actin stained with rhodamine phalloidin, red). Attached objects (bacteria or beads) per cell and % cells with stress fibers were determined from images taken at indicated timepoints (C, F, I). Data shown are means ± standard deviation from twelve images (4 frames from n = 3, representing at least 100 cells/experimental condition). *V. parahaemolyticus* CAB4ΔMAM7 or *E. coli* BL21-MAM7ΔN_1–44_ (MAM retained in the cytoplasm), (B, E, bacteria in green) or of polymer beads coupled to GST only (H, beads in blue) to Hela cells did not cause changes in the actin phenotype. Images shown are of 1 hour time points and are representative of a set of three experiments. Bar, 10 µm. G-actin (blue) and F-actin (red) content of cells treated with MAM (M) or controls (C) was quantified at 1 hour post treatment (J) and compared to serum-starved, untreated cells (−) and cells treated with F-actin enhancing solution (+). Results are means ± s.e.m. (n = 2) and (*) indicates statistical significance (p<0.05 in a student's two-tailed unpaired t-test).

**Figure 2:**
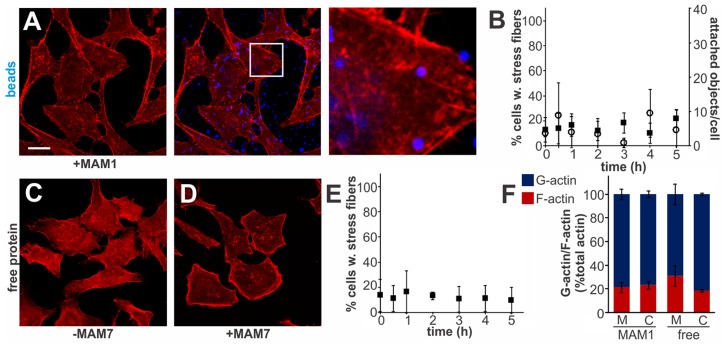
Multivalent, clustered MAM7 is necessary to trigger actin rearrangements in host cells. Attachment of polymer beads coupled to GST-MAM1 (1 mce domain from MAM7, A) or attachment of soluble GST (C) or GST-MAM7 (D) did not cause significant changes in actin phenotype. Attached objects (beads) per cell and % cells with stress fibers were determined from images taken at indicated timepoints (B, E). Data shown are means ± standard deviation from twelve images (4 frames from n = 3). Images shown are of 1 hour time points and are representative of a set of three experiments. Bar, 10 µm. G-actin (blue) and F-actin (red) content of cells treated with MAM (M) or controls (C) was quantified at 1 hour post treatment. Results are means ± s.e.m. from duplicate experiments (F).

Multivalent Adhesion Molecule (MAM) 7 present on the outer membrane of *V. parahaemolyticus* mediates attachment of bacteria to host cells [14]. We used *V. parahaemolyticus* strain CAB4 to study the infection phenotype in Hela cells. CAB4 is derived from the well characterized, pathogenic RIMD2210633 strain [26], but lacks both thermostable hemolysins (Δ*tdhA* Δ*tdhS*) and does not express the two type III secretion systems (Δ*exsA* Δ*vtrA*). Despite lacking known virulence factors, infection with *V. parahaemolyticus* CAB4 strain caused pronounced cytoskeletal changes, with thick strands of filamentous actin forming (Fig. 1A). The appearance of F-actin fibers was observed almost immediately upon infection and persisted throughout the course of the experiment (Fig. 1C). In contrast, no changes in the actin phenotype were observed in cells infected with CAB4Δ*vp1611* lacking MAM7 (Fig. 1B). As such, MAM7 is necessary to trigger the observed actin rearrangements upon infection with *V. parahaemolyticus* CAB4. Next, we investigated if MAM7 is sufficient to cause actin stress fiber formation in Hela cells. Heterologous surface-expression of *V. parahaemolyticus* MAM7 in otherwise non-adherent *Escherichia coli* is sufficient to mediate their attachment to a wide range of host cells [14]. Infection of cells with this recombinant, attaching *E. coli* strain recapitulated the same sustained actin rearrangements seen upon infection with CAB4 (Fig. 1D, F). In contrast, expression of translocation-deficient MAM7 (MAM7ΔN_1–44_) in *E. coli* lead to only low levels of attachment and did not trigger actin rearrangements (Fig. 1E). This demonstrates that *V. parahaemolyticus* MAM7 is necessary and sufficient to convey upon non-pathogenic bacteria the ability to attach to host cells and trigger actin rearrangements. Next, chemical cross-linking was used to directionally couple purified MAM7 protein to the surface of fluorescent polymer beads, thereby mimicking exposure of the adhesin on the bacterial surface. We used this “bacteriomimetic” system to study the effect of MAM7 on host cells independent of other bacterial molecules. Beads directionally coupled to the N-terminus of a protein containing all seven mammalian cell entry (mce) domains of *V. parahaemolyticus* MAM7 (GST-MAM7) attach to host cells and trigger sustained actin rearrangements, mimicking the phenotype seen upon infection with CAB4 (Fig. 1G, I). In contrast, beads coupled to GST alone did not significantly bind to host cells and caused no actin rearrangements (Fig. 1H). Beads coupled to protein containing only a single mce domain (MAM1) also failed to be recruited to the host cell surface in high numbers and did not cause changes in cytoskeletal organization (Fig. 2A, B). Free, soluble, uncoupled MAM7 or free GST also did not cause any cytoskeletal reorganization (Fig. 2C–E). The visually observed changes in actin phenotype were also recapitulated using quantitative analysis of cellular G-actin and F-actin contents by fractionation of lysates, Western Blotting and densitometry (Fig. 1J and 2F). We conclude that *V. parahaemolyticus* MAM7, through multivalent binding of host receptors and when clustered on the host cell surface, causes sustained rearrangements in the actin cytoskeleton, visible as bundles of F-actin.

### Clustered MAM7 triggers actin rearrangements through RhoA activation

**Figure 3:**
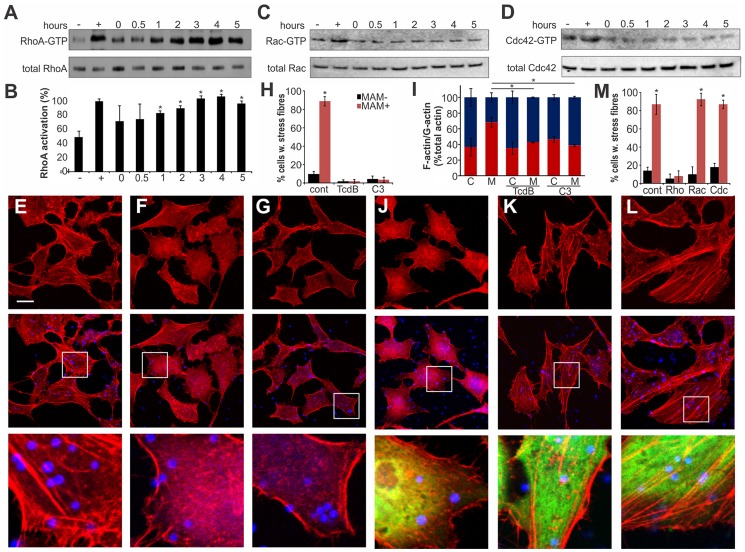
Clustered MAM7 triggeres actin rearrangements through RhoA activation. Following incubation of Hela cells with bead-MAM7, RhoA-GTP levels were determined and compared to total RhoA levels either immediately, 30 min, 1, 2, 3, 4 and 5 hours following bead attachment (A). RhoA activation in percent (B) was determined from ratios of band intensities of RhoA-GTP/total RhoA, with the positive control (GTPγS-incubated sample) set to 100%. Negative control (−) were cells incubated with bead-coupled GST for 5 hours. Results are means ± standard deviation, n = 3. Data points significantly different from the negative control (p<0.05 as determined by two-tailed unpaired student's t-test) are indicated (*). Experiments described in (A) were repeated to detect activated and total Rac (C) and Cdc42 (D). To test whether GTPase inactivation would affect actin phenotypes, cells were either left untreated (E), treated with TcdB (F) or cell-permeable C3 transferase (G) prior to attachment of bead-MAM7. % cells with stress fibers (H) were determined from image analysis following experiments with untreated (cont), TcdB- or C3-treated cells and attachment of both bead coupled GST-MAM7 (MAM+, red bars) or coupled GST (MAM−, black bars) and data are means ± standard deviation from 12 images (4 frames from n = 3). F-actin/G-actin content was determined from the same samples (C, cont. beads; M, MAM beads) (I). Results are means ± s.e.m. from duplicate experiments and significantly different data are marked (*). Cells transfected with DN EGFP-RhoA (J), DN EGFP-Rac (K) or DN EGFP-DNCdc42 (L) were attached to bead-coupled GST-MAM7 and cells stained with rhodamine-phalloidin. Images shown are of 1 hour post bead attachment and are representative of a set of three experiments. Bar, 10 µm. % cells with stress fibers (M) were determined from image analysis following experiments on cells transfected with pcDNA3-EGFP (cont), DN RhoA, DN Rac or DN Cdc42 and attachment of both bead coupled GST-MAM7 (MAM+, red bars) or coupled GST (MAM−, black bars) and data shown are means ± standard deviation from 12 images (4 frames from n = 3).

Actin rearrangements are generally mediated by activation of small GTPases RhoA, Rac and/or Cdc42. We tested the activation levels of all three GTPases by studying the fraction of GTP-bound proteins over time, following binding of MAM7-beads to host cells (Fig. 3). We observed a sustained activation of RhoA, but not Rac or Cdc42, which persisted over several hours in the presence of cell-bound MAM7 beads (Fig. 3A–D). To analyze if actin rearrangements following MAM7 attachment would be dependent on RhoA, Rac or Cdc42, we treated cells with *Clostridium difficile* toxin B (TcdB) or *C. botulinum* C3 transferase. TcdB irreversibly deactivates Rho GTPases by glycosylation of the catalytic threonine residue. C3 selectively inactivates RhoA, B and C but not Rac or Cdc42 by ADP-ribosylation of asparagine 41 in the effector region [27]. While untreated cells displayed stress fibers when incubated with fluorescent MAM7 beads, no actin rearrangements where observed in cells pre-treated with either TcdB or C3 transferase (Fig. 3E–H). The observed change in actin phenotype was also confirmed by quantification of cellular G-actin and F-actin (Fig. 3I). We also studied the effect of MAM7 binding on cells overexpressing either dominant negative RhoA, Rac or Cdc42. Expression of RhoAT19N-GFP abolished actin rearrangements, while expression of either RacT17N-GFP or Cdc42T17N-GFP had no effect (Fig. 3J–M). We conclude that binding of multivalent, surface-coupled MAM7 to the host cell membrane specifically activates RhoA, which in turn triggers the observed actin rearrangements.

### MAM7-mediated actin rearrangements proceed via the ROCK/LIM-kinase/cofilin signaling axis

**Figure 4:**
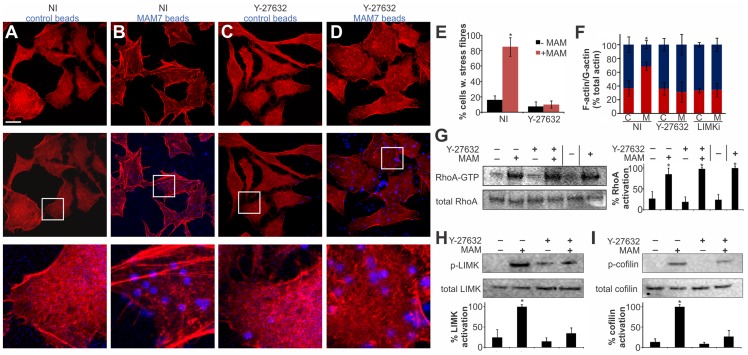
MAM-mediated actin rearrangements proceed via the ROCK/LIMK/cofilin signaling axis. Hela cells were incubated with bead-coupled GST (A, C) or GST-MAM7 (B, D) for 1 hour following mock treatment (A,B) or treatment of cells with 10 mM Y-27632 for 60 minutes (C, D) and F-actin stained with rhodamine-phalloidin (red). % cells with stress fibers (E) were determined from image analysis following mock treatment (NI) or Y-27632 treatment of cells and attachment of bead coupled to GST-MAM7 (+MAM, red bars) or coupled to GST (−MAM, black bars). Data shown are means ± standard deviation from twelve images (4 frames from n = 3). Images are representative of a set of three experiments. Bar, 10 µm. F-actin/G-actin content was determined for the same experimental conditions (C, cont. beads; M, MAM beads), (F). Results are means ± s.e.m. from duplicate experiments. RhoA activation levels were determined under the same experimental conditions and data significantly different from the negative control are marked (*), (G). % activation was determined by densitometry and samples statistically significantly different from the negative control (GDP-lysate, −) are indicated (*). Levels of p-LIMK and total LIMK (H) or p-cofilin and total cofilin (I) were determined by Western Blotting and densitometry. % activation was determined as ratios of band intensities from phosphorylated and total protein and normalized to mock treated cells attached to bead coupled GST-MAM7 (100%). Data shown are means ± standard deviation from at least three independent experiments and data statistically significantly different from the negative control (student's unpaired two-tailed t-test, p<0.05) are indicated (*).

Several cellular pathways are involved in relaying signaling between activated RhoA and the actin cytoskeleton and the observed actin rearrangements could be a result of either increased stress fiber formation or a decrease in actin depolymerization [28]. We tested if the MAM-induced RhoA activation and ultimately actin rearrangements proceed via the Rho-associated serine/threonine kinase ROCK, a downstream effector of RhoA, by treating cells with the ROCK inhibitor Y-27632 [29]. Cells incubated with control beads showed no perturbation in the actin cytoskeleton, either in the presence or absence of Y-27632 (Fig. 4A, C, E). In contrast, cells incubated with bead-coupled MAM displayed stress fibers but this phenotype was almost completely abolished in Y-27632 treated cells (Fig. 4B, D, E). These findings were recapitulated when we quantified the cellular G-actin and F-actin content under identical experimental conditions (Fig. 4F).

Next, we tested whether MAM-induced ROCK activation takes place upstream or downstream of RhoA. We analyzed RhoA activation in the presence and absence of MAM beads, either on untreated or Y-27632 treated cells. These data show that when ROCK is inhibited, even though MAM-induced stress fiber formation is abolished, RhoA activation levels remain high (Fig. 4G). We thus conclude that MAM-induced ROCK activation occurs downstream of RhoA.

Next, we tested the activation of LIM kinase (LIMK) and cofilin, two key signaling proteins downstream of ROCK. A significant fraction of LIMK was phosphorylated in the presence of MAM-beads, but the p-LIMK level was much reduced if cells were pre-treated with Y-27632 prior to MAM7 bead adhesion. Incubation with either control beads alone or in combination with Y-27632 treatment did not cause significant LIMK phosphorylation (Fig. 4H). LIMK activation causes phosphorylation of cofilin and thus inhibition of its actin depolymerization activity. We observed an increase in p-cofilin in cells with attached MAM-beads, which was abolished by Y-27632 treatment prior to attachment. Incubation with either control beads alone or following Y-27632 treatment did not cause significant changes in p-cofilin levels (Fig. 4I). In addition, treatment of cells with LIMK inhibitor prior to MAM adhesion lead to a loss of the actin phenotype and concurrent loss of increased F-actin contents (Fig. 4F). We conclude that MAM-induced actin rearrangements proceed via the RhoA/ROCK/LIM-K/cofilin pathway and are the result of abrogated actin depolymerization rather than de novo polymerization.

### RhoA activation depends on MAM7 interaction with phosphatidic acids and is independent of the co-receptor fibronectin

**Figure 5:**
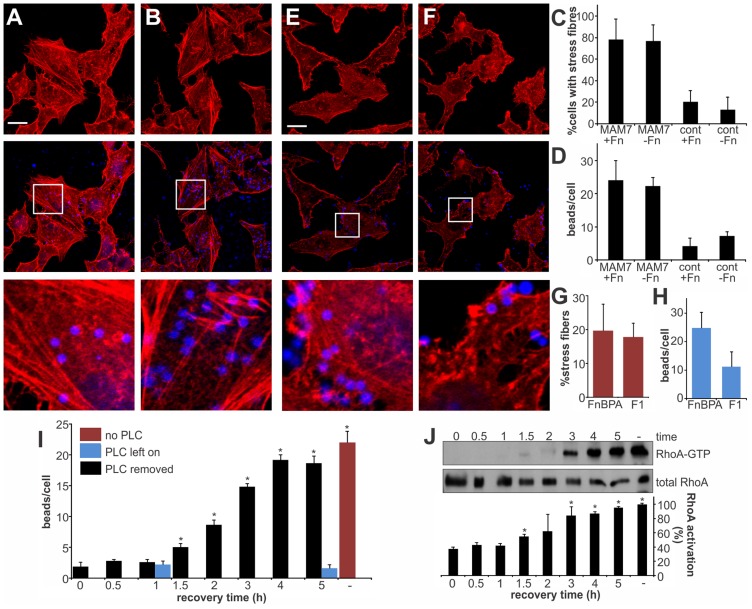
RhoA activation depends on MAM binding to phosphatidic acids and is independent of the co-receptor fibronectin. Hela cells were incubated with bead-coupled GST-MAM7 for 1 hour either without (A) or with (B) prior masking of MAM binding epitopes on fibronectin with antibodies and F-actin stain with rhodamine-phalloidin. % cells with stress fibers (C) were determined from image analysis following treatment with non-specific antibody (+Fn) or treatment of cells with α-Fn antibody, thereby masking the MAM binding epitope on fibronectin (−Fn) and attachment of bead coupled to GST-MAM7 (MAM7) or coupled to GST (cont). Number of attached beads per cells was unchanged by treatment with α-Fn compared to control antibody (D). Hela cells were incubated with bead-coupled FnBPA FnBR1-11 (E) or F1 FUD (F) and stained with rhodamine-phalloidine (red). % cells with stress fibers (G) and number of attached beads per cell (H) were determined from image analysis following both treatments. Data shown are means ± standard deviation from twelve images (4 frames from n = 3). Images shown are of 1 hour time points and are representative of a set of three experiments. Bar, 10 µm. Beads attached per cell (I) and RhoA activation (J) were determined either with no prior treatment of cells (−, red bar), under constant treatment with PLC (blue bars) or after treatment of cells with PLC, removal of PLC and attachment of bead-coupled GST-MAM7 for the indicated time points (0–5 hours post recovery, black bars). RhoA activation was determined as the ratio of band intensities for RhoA-GTP and total RhoA and normalized to samples without PLC treatment and following 5 hours of attachment of GST-MAM7 beads (−, 100% activation). Data statistically significantly different from t = 0 (I) or the negative control (J) as per student's unpaired two-tailed t-test, p<0.05, are indicated (*).

We have previously shown that MAM7 interacts with two types of receptors in the host cell membrane. Each of the seven mce domains within MAM7 is capable of interacting with a phosphatidic acid phospholipid molecule, thereby mediating high affinity binding of bacteria to host cells. Recognition of fibronectin is achieved by a repeat of at least five mce domains and while this interaction is dispensable for attachment, it increases the on-rate of bacterial binding to host cells [15]. We asked if the observed actin rearrangements are a result of MAM binding to fibronectin or phosphatidic acid receptors on host cells, or both. We made MAM attachment to host cells independent of binding to fibronectin by blocking the MAM binding epitope on fibronectin with an antibody [15]. This way, binding of MAM7 to host cells was only mediated by phosphatidic acid receptors. Cells either pre-treated with α-Fn antibodies or non-specific control antibodies were incubated with MAM7 beads or control beads. Following incubation with MAM7 beads, stress fibers were observed in both cells treated with control antibodies (+Fn), or α-Fn antibodies (−Fn), (Fig. 5A–C). In contrast, no actin changes were observed in cells following treatment with either antibody followed by control beads (Fig. 5C). As previously described, uncoupling MAM7 binding from its co-receptor fibronectin did not change the overall number of beads bound per cell if sufficient time was allowed for attachment (Fig. 5D). The interaction between fibronectin and MAM has been mapped to the N-terminal region of fibronectin, which is an epitope commonly exploited by bacterial adhesins for binding [30]. Both *Staphylococcus aureus* fibronectin binding protein A (FnBPA) and *Streptococcus pyogenes* protein F1 bind the N-terminal part of fibronectin with high affinity [31], [32]. Thus, we tested whether portions of these two adhesins sharing the same binding epitope with MAM would cause similar actin rearrangements to those observed with MAM7. We incubated cells with beads coupled to the fibronectin-binding region of either FnBPA (FnBR1-11) or F1 (FUD), as previously described [33]. Although both preparations bound to cells with high efficiency, neither caused stress fiber formation (Fig. 5E–H). Taken together, these findings strongly suggest that fibronectin is not involved in the observed signaling pathway between MAM7, RhoA and actin. To see whether changes in the membrane lipid composition would impact MAM's ability to trigger RhoA activation, we treated cells with phospholipase C (PLC). MAM7 beads were added to cells either immediately or up to five hours following PLC treatment and subsequent enzyme removal, and levels of beads per cell as well as RhoA activation were measured. In untreated cells, approximately 23 beads were bound per cell (Fig. 5I, red bar). No bead binding was observed if cells were continuously exposed to PLC, since the interaction with fibronectin alone is insufficient to mediate binding (Fig. 5I, blue bars). If PLC was removed, the interaction between lipid receptors and MAM7, and thus bead binding, was initially completely abolished but was gradually recovered until normal binding levels were regained after four hours of recovery (Fig. 5I, black bars). A similar time course was established for RhoA activation upon MAM bead attachment, with full GTPase activation recovered four hours after removal of PLC (Fig. 5J). We conclude that the MAM7 co-receptor fibronectin is dispensable not just for MAM7 binding, but also for the subsequent activation of RhoA and actin rearrangements caused by adhesion. The observed signaling cascade thus depends on the interaction of multivalent, surface-clustered MAM7 adhesins with phosphatidic acid lipids in the host cell membrane.

### MAM adhesion is necessary and sufficient to disrupt epithelial barrier function and promote bacterial transmigration

**Figure 6:**
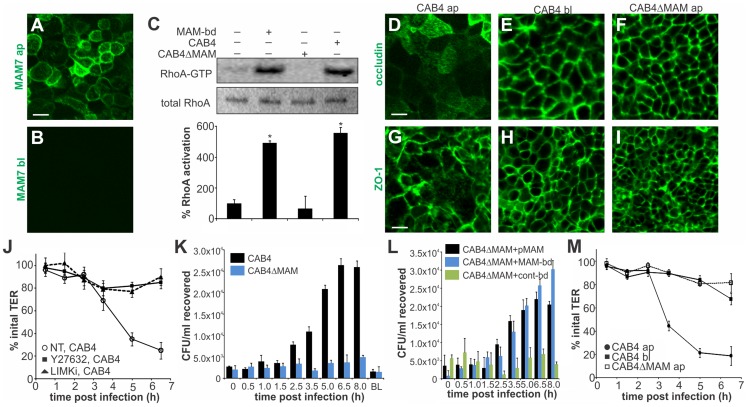
MAM adhesion is necessary and sufficient to disrupt epithelial barrier function and promote bacterial transmigration. Purified GST-MAM7 was added to the apical (ap, A) or basolateral (bl, B) compartment of polarized Caco-2 layers and incubated for 2 hours. Samples were imaged by immunofluorescence microscopy using anti-GST and FITC-labeled secondary antibodies. Images shown are representative of a set of twelve images (4 frames from n = 3). Polarized Caco-2 layers were either left untreated or incubated with bead-coupled GST-MAM7 (MAM-bd), CAB4 or CAB4ΔMAM for two hours and Rho activation levels were determined as ratio of band intensities from RhoA-GTP and total RhoA (and normalized to untreated layers, 100% activation, C). Results significantly different from untreated are marked (*, n = 3). Polarized Caco-2 layers were infected with CAB4 from the apical side (D, G), CAB4 from the basolateral side (E, H) or CAB4ΔMAM from the apical side (F, I). Cells were immunostained with anti-occludin (D–F) or α-ZO-1 (G–I) and FITC-labeled secondary antibody. Images shown are representative of a set of 12 images (4 frames from n = 3). Transepithelial electrical resistance (TER) was measured following infection of polarized Caco-2 layers with CAB4 without (NT, white circles) or with prior treatment of cells with Y-27632 (black squares) or LIMKi (black triangles), (J). Bacterial recovery from the basolateral compartment following apical infection or from the apical compartment after 8 hours of basolateral infection (BL) was determined for CAB4 (black bars) or CAB4ΔMAM (blue bars) added at an MOI of 100 (K). Bacterial recovery from the basolateral compartment following apical infection for the indicated time points with CAB4ΔMAM reconstituted with a plasmid expressing MAM7 (black bars) or infected with a mixture of CAB4ΔMAM and bead-coupled GST-MAM7 (blue) or bead-coupled GST only (green), (L). TER was measured on polarized Caco-2 layers infected with CAB4 apically (black circles), CAB4 basolaterally (black squares) or CAB4ΔMAM apically (white circles) and normalized to basal TER prior to infection (100%), (M). Data shown in J-M are means ± standard deviation (n = 3).

*Vibrio parahaemolyticus* mostly causes gastroenteritis and on rare occasions it can lead to systemic disease in immunocompromised patients. To better reflect the *in vivo* situation, we studied the effect of MAM on polarized intestinal epithelial (Caco-2) cells. Differentiated Caco-2 monolayers are a good model of the epithelium in the small intestine, the main site of *V. parahaemolyticus* infection. When grown on permeable supports, Caco-2 cells form monolayers with well differentiated brush border microvilli and properties resembling those of the small intestinal epithelium [34]. First, we studied the localization of MAM7 on polarized cell layers. MAM7 exclusively bound to the apical side of the epithelial layer, with the protein being enriched at cellular junctions (Fig. 6A). No binding was observed when MAM protein was added to the basolateral side (Fig. 6B). Similar to the effects seen in Hela cells, MAM-coupled beads and *V. parahaemolyticus* CAB4, but not a MAM deletion strain (CAB4ΔMAM), caused a significant increase in RhoA activation compared to untreated cells (Fig. 6C). Because MAM7 was enriched at cell junctions and RhoA activation is capable of affecting the distribution of tight junction proteins, we studied the localization of tight junction markers during infection with *V. parahaemolyticus*. Apical infection with CAB4 caused a re-distribution of the tight junction markers occludin and zonula occludens protein 1 (ZO-1) (Fig. 6D, G). In contrast, the distribution of both tight junction proteins remained unchanged when cells were infected with CAB4 from the basolateral side (Fig. 6E, H) or apically with the MAM knockout strain CAB4ΔMAM (Fig. 6F, I).

Next, we asked if re-distribution of tight junction proteins during infection would affect epithelial barrier function. When CAB4 was added to the apical surface of a differentiated layer, a marked decrease in transepithelial electrical resistance (TER) was observed approximately three hours post infection. This change was mediated via ROCK/LIMK activation, since treatment of cells with either Y-27632 or LIMK inhibitor abolished the CAB4-mediated decrease in TER (Fig. 6J). Similarly, no significant decrease in TER was observed up to seven hours post infection with either CAB4ΔMAM added apically or CAB4 added to the basolateral side of the epithelium (Fig. 6M).

We also investigated whether the disruption of cell-cell junctions was sufficient to allow for bacterial transmigration. Polarized cells were infected with either CAB4 or CAB4ΔMAM and bacterial titers in the opposing compartment were determined either immediately or up to eight hours post infection. When either CAB4 or CAB4ΔMAM were added to the basolateral side, no bacteria were recovered on the apical side. In contrast, CAB4 was recovered from the basolateral side following infection from the apical side. Bacterial numbers on the basolateral side increased significantly 2.5 hours post infection and continued to increase until 6.5 hours post infection, reaching approximately 1% of the initial infecting population. In epithelial layers apically infected with CAB4ΔMAM, no bacteria were detected on the basolateral side (Fig. 6K). The loss of MAM could be compensated either by the expression of MAM in trans or by treatment of cells with bead-bound MAM, but not with control beads (Fig. 6L). We concluded that MAM selectively binds to the apical side of polarized intestinal epithelial cells, causing a re-distribution of tight junction proteins, disruption of barrier integrity and bacterial transmigration.

### MAM7 function accelerates T3SS1-mediated lysis of polarized epithelial cells

**Figure 7:**
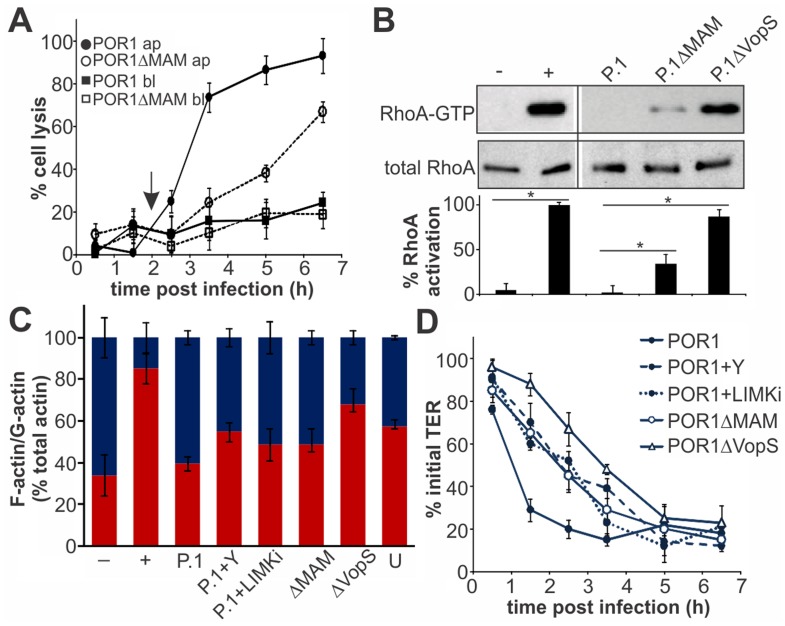
MAM function accelerates T3SS1-mediated lysis of polarized epithelial cells. Cell lysis (A) was determined following infection of Caco-2 layers with POR1 apically (black circles) or basolaterally (black squares) or POR1ΔMAM apically (white circles) or basolaterally (white squares). Results were normalized to Triton-induced cell lysis (100%) and uninfected cells (0%). Arrow (2 hrs) indicates time point chosen for experiments shown in (B) and (C). Data shown are means ± standard deviation (n = 3). Polarized Caco-2 layers were infected with POR1 (P.1), POR1ΔMAM or POR1ΔVopS for 2 hrs and Rho activation levels were determined as the ratio of band intensities from RhoA-GTP and total RhoA (and normalized to GTPγS-treated lysate, (+, 100% activation). Neg. control (−): GDP-treated lysate. Statistical significance is indicated (*, n = 2), (B). F-actin and G-actin content was determined in polarized Caco-2 monolayers after serum starvation (−), treatment with F-actin enhancing solution (+), infection with POR1, POR1 after treatment with Y-27632 (P.1+Y), with LIMK inhibitor (P.1+LIMKi) or infected with POR1ΔMAM or POR1ΔVopS for 2 hrs, or on untreated cells (U). Results are means ± s.e.m. (n = 2), (C). Transepithelial electrical resistance (TER) was measured on polarized Caco-2 layers infected with POR1, POR1 after treatment with Y-27632 or after treatment with LIMK inhibitor or infected with POR1ΔMAM or POR1ΔVopS (D) and normalized to basal TER prior to infection (100%). Results are means ± standard deviation (n = 3).

Finally, we asked if the epithelial disruption caused by MAM-mediated adhesion would contribute to infection in a virulent strain. Polarized intestinal epithelium was infected with the virulent strain POR1 or POR1ΔMAM from the apical or basolateral side. Infection with POR1 apically lead to cytotoxicity and rapid cell lysis, with almost complete cell death five hours post infection (Fig. 7A). The cytotoxicity profile was significantly delayed upon infection with POR1ΔMAM and cell death reached only approximately 70% even seven hours post infection. When cells were infected with either POR1 or POR1ΔMAM from the basolateral side, no significant increase in cytotoxicity was observed over the course of the experiment (up to seven hours post infection). POR1 contains the T3SS effector VopS, which causes RhoA inhibition by irreversible AMPylation of a threonine residue in the switch I region [9]. Thus, we investigated the contribution of MAM to the overall RhoA activation levels in polarized Caco-2 cells infected with the virulent strain. At 2 hours post infection, prior to the onset of cell lysis, RhoA activity was completely abolished in POR1 infected cells. In contrast, RhoA was highly activated in POR1ΔVopS. An intermediate level of RhoA activation was observed in cells infected with POR1ΔMAM (Fig. 7B). We also analyzed the G-actin and F-actin content of polarized Caco-2 cells 2 hours post infection. Within 2 hours, POR1 infection lead to a drop in F-actin content compared to untreated cells, which was mediated by the activity of VopS. In the absence of MAM, or in the presence of ROCK- or LIMK inhibitors, the F-actin content was higher compared to POR1 infected cells (Fig. 7C). Finally, we measured the transepithelial resistance in Caco-2 monolayers infected with the virulent strain. POR1 caused a rapid decrease of TER, which was markedly slowed by treatment of cells with Y-27632 or LIMK inhibitor. Similarly, both POR1ΔMAM and POR1ΔVopS showed a slight delay in depolarization (Fig. 7D).

## Discussion

Previously, we reported that *V. parahaemolyticus* Multivalent Adhesion Molecule (MAM) 7 and several of its homologs from other Gram-negative enteric pathogens mediate initial attachment of bacteria to host cells [14]. In this study, we demonstrated that clusters of multivalent MAM molecules, by binding to the host cell membrane, facilitate activation of the host small GTPase RhoA, which in turn leads to actin rearrangements. Clustering of MAMs is achieved by nature, through display of multiple adhesion molecules on the bacterial outer membrane [14], but can be mimicked by coupling recombinant MAM molecules to a polymer bead with roughly the same dimensions as a bacterium. Soluble MAM failed to achieve the same effect on host cell signaling.

MAMs interact with host cells via two cellular receptors, the protein fibronectin and the phosphatidic acid (PA) phospholipids. While the former is a well-characterized pathogen receptor [30], [35], [36], direct binding of a bacterial adhesin to a host cell lipid is a new paradigm of host-pathogen interaction. Over recent years, manipulation of cellular lipids by pathogens has been an emerging field of study, and it has become evident that host cellular lipids are often a primary target of bacterial virulence factors [11], [37], [38]. Herein, we showed that MAM's impact on RhoA activation is mediated through its interaction with phosphatidic acid lipids in the host membrane and that its co-receptor fibronectin is dispensable for its function as a signaling effector. Taken together, these findings suggest a mechanism whereby the interaction of clustered MAM adhesins with host membrane lipids causes rearrangements of the latter and that this acts as a signal leading to RhoA activation. However, direct observation of such hypothesized rearrangements of phosphatidic acid molecules within the host membrane on the nanoscale is not within the scope of our studies but is an intriguing possibility and something we are currently investigating.

We have elucidated the signaling pathway downstream of RhoA and show the MAM-triggered signal is relayed from activated RhoA, via the Rho-associated serine/threonine kinase ROCK and LIM kinase, to result in phosphorylation of cofilin. Cofilin is an actin-binding protein which mediates actin depolymerization [39]. Its interaction with actin and thus its depolymerization activity is disrupted by phosphorylation, resulting in a net stabilizing effect on filamentous actin and apparent increase in actin stress fibers. Although a large part of our experiments was performed on Hela cells because changes in the actin phenotype following serum starvation are visually easier discernible in this cell type, we show that the MAM-mediated effect on actin also proceeds via ROCK and LIMK activation in polarized intestinal epithelial cells, a more relevant system for studies on *V. parahaemolyticus*. Since we observe MAM-induced RhoA activation also in polarized epithelial cells, we hypothesize that this RhoA activation facilitates subsequent activation of the ROCK/LIMK/cofilin signaling axis, however we cannot show whether RhoA activation is required in this model, since RhoA inactivation itself leads to increased transepithelial permeability [40].

In the polarized epithelial system, MAM7 selectively attached to the apical side of the layer and attachment caused a marked redistribution of tight junction proteins. A similar phenotype has been described to occur following infection of epithelial cells with other pathogens, such as enteropathogenic *E. coli* (EPEC) or the protozoan parasite *Giardia lamblia*. With EPEC infection, paracellular permeability also resulted from a redistribution of tight junction proteins upon RhoA activation, although in that case RhoA activation has been largely attributed to the activities of type III system-secreted effectors [41], [42]. In *G. lamblia*, barrier failure was attributed to apoptosis of enterocyes [43]. Activation of RhoA through the establishment of a signaling complex consisting of bacterial adhesin clusters and host membrane lipids on the host cell surface is, to our knowledge, a previously unrecognized strategy to achieve epithelial barrier disruption.

We demonstrated that the action of MAM7 causes epithelial barrier disruption, as evidenced both by a decrease in transepithelial resistance and the ability of bacteria to transmigrate to the basolateral side of the epithelium. It has previously been shown that CAB4 is unable to invade epithelial cells [12], so this is likely the result of bacteria moving through compromised cell-cell junctions. It has been shown previously that epithelial integrity is compromised following *V. parahaemolyticus* infection, both in cultured polarized epithelial cells and *in vivo*. Animal infection models have shown increased transepithelial permeability using fluorescent dextran as a tracer, but the effect was not attributed to any particular virulence factor [6]. Earlier experiments on polarized Caco-2 cells demonstrated a similar effect on epithelial integrity and ruled out TDH and TRH toxins as the culprit [13]. A comparison between *V. parahaemolyticus* clinical isolates and environmental strains implicated T3SS2 in transepithelial permeability. However, no whole genome sequences are available for the strains used in this study and we therefore do not know if they encode for a MAM homolog and if so, to what extent it would share sequence similarity to RIMD2210633 MAM7 (*vp1611*) [44]. More recent studies on Caco-2 and mixed M cell-like co-cultures demonstrated that T3SS1 does not significantly contribute to translocation, while T3SS2 is dispensable but has a moderately enhancing effect on translocation in a RIMD2210633 background [45]. Herein we show that MAM7 is sufficient to cause barrier disruption in cultured polarized epithelium. In the context of a T3SS-competent, virulent strain, MAM induces transepithelial permeability and depolarization of the epithelium early during infection. Since MAM is constitutively expressed and present at the early stages of infection, its effect takes hold almost immediately and RhoA activation is detectable as early as 30 minutes post infection (the earliest time point measured here). The resulting depolarization and disruption of cell-cell junctions leads to an increase in host cell surface available for translocation of type III secreted bacterial effectors. Overall, this mechanism accelerates effector-mediated functional changes in host cells, such as VopS-mediated irreversible RhoA inactivation and concomitant actin depolymerization, thus speeding up infection. These findings strongly indicate experiments comparing the effect of wild type and MAM knockout strains in an animal model and this should be the next step to show if indeed MAM contributes to transepithelial permeability and infection *in vivo*.

Overall, the study we present here demonstrated that the contribution of *Vibrio parahaemolyticus* MAM7 to the pathogen's infection profile is not limited to its function in early bacterial attachment. By establishing signaling complexes consisting of clustered MAM adhesins and host membrane lipid receptors on the host cell surface, it additionally acts as an effector of host cellular GTPase signaling and its action culminates in breaching of the epithelial barrier. This is, to our knowledge, a previously unrecognized strategy by which a bacterial pathogen disrupts intestinal epithelial function and the detailed molecular mechanism of how this is achieved certainly deserves our further investigation.

## Materials and Methods

### Bacterial strains and growth conditions

The construction of BL21-MAM7, BL21-MAMΔN_1–44_, CAB4, POR1, POR1ΔMAM (POR1Δ*vp1611*) and POR1ΔVopS has been described elsewhere [9], [12], [14]. The *V. parahaemolyticus* MAM deletion strain CAB4Δ*vp1611* was constructed using the same method and same vector construct (pDM4 containing regions 1 kb up- and downstream of *vp1611*) described in these references. Strains were grown on MLB (*V. parahaemolyticus*) or LB agar (*E. coli*), with 100 µg/ml of kanamycin or ampicillin added for selection where required.

### Cell culture conditions and polarization of epithelial cells

HeLa and Caco-2 epithelial cell lines were cultured at 37°C and under 5% CO_2_ in Dulbecco's Modified Eagle Medium (DMEM) containing 10% heat-inactivated fetal bovine serum, 4500 mg/L glucose, 0.5 mM L-glutamine, 100 units/ml penicillin and 20 µg/ml streptomycin. For GTPase activation and microscopy assays, cells were serum-starved for 40 hours prior to treatment. For infection experiments, DMEM with no added antibiotics was used. For experiments on polarized Caco-2 cells, cells were seeded on polycarbonate 3.0 µm pore size transwell filters (Costar) at 200000 cells/ml. Cells reached confluency after approximately 5–6 days, at which point several transepithelial resistance (TER) measurements were taken to check the integrity of the layer and establish baseline measurements. TER measurements before and during infection experiments were taken with a Millicell-ERS resistance apparatus (Millipore).

### Chemical coupling of proteins to beads

Expression and purification procedures for recombinant proteins have been described in detail elsewhere (see [14] for GST-MAM7 [15], for GST-mce1 and [33] for GST-FnBPA FnBR1-11 and F1 FUD constructs). Purified proteins were immobilized on amine modified fluorescent blue polystyrene beads with a mean diameter of 2 µm (Sigma) using Sulfo-SMPB (sulfosuccinimidyl 4-[*p*-maleimidophenyl]butyrate) cross-linking under reducing conditions, as outlined in the manufacturer's protocol (Pierce). Bead-coupled proteins were added to experiments to give a final concentration of 500 nM immobilized protein and a surface density of 1.5×10^5^ molecules per bead (giving a spacing of approximately 57 nm).

### Attachment and infection experiments

Tissue culture cells were washed with PBS (phosphate-buffered saline) prior to the addition of bacteria in tissue culture medium without antibiotics. Bacteria were added to give a multiplicity of infection (MOI) of 100, except for POR1 and derivatives, where an MOI of 10 was used. Plates were centrifuged (1000×g, 22°C, 5 minutes) prior to incubation at 37°C for 30 minutes to eight hours, depending on the experiment. To uncouple MAM binding from fibronectin or phosphatidic acid, respectively, cultured cells were incubated with anti-Fn antibody (Sigma, 50 µg/ml in PBS) or treated with 50 µg/ml phospholipase C (Sigma) in PBS for 15 min prior to infection, as previously described [15]. For enumeration of bacteria, samples were removed at time points as indicated and were serially diluted, plated on agar plates, incubated at 37°C for sixteen hours and CFU counts determined the following day. For cytoxicity assays, 200 µl of culture supernatant was removed in triplicate from each well at timepoints as indicated, centrifuged (1000×g, 22°C, 5 minutes), and 100 µl of the supernatant transferred to a fresh 96-well plate for assays. To quantitate cell lysis, the amount of lactate dehydrogenase (LDH) released into the culture medium was determined using the LDH cytotoxicity detection kit (Takara) according to the manufacturer's instructions. Results are presented in % lysis, relative to negative (uninfected) and positive (Triton X-100 lysed cells) controls.

### Transfection and immunofluorescence microcopy

Cells were transfected with pcDNA3 containing either EGFP, EGFP-RhoAT19N, EGFP-RacAT17N or EGFP-Cdc42T17N using Fugene HD (Roche) transfection reagent according to the manufacturer's protocol. For microscopy, cells were fixed with 3.2% formaldehyde, permeabilized with 0.1% Triton X-100 and stained with rhodamine-phalloidin to visualize F-actin and SYTO-13 to visualize DNA as indicated. For immunofluorescence microscopy, we used α-GST, α-occludin and α-ZO-1 antibodies (Sigma) diluted 1∶500, followed by FITC-labeled α-rabbit antibody (Sigma) at a 1∶1000 dilution. Images were taken either on a Zeiss LSM 510 scanning confocal microscope or a Nikon Eclipse Ti fluorescence microscope and images were prepared using ImageJ and Corel Draw X5. For quantification of the F-actin phenotype, the total number of cells as well as number of cells containing stress fibers, were enumerated. Some fields contained cells displaying cortical actin, however this phenotype was observed across experiments and was independent of MAM adhesion. Thus, these cells were not counted as positive. Data shown are means ± standard deviation from twelve images (four frames from triplicate experiments, representing at least 100 cells/experimental condition).

### Western blotting and antibodies

Proteins were separated by SDS-PAGE and transferred onto nitrocellulose membrane. Membranes were blocked with 5% BSA in TBS-T (Tris-buffered saline containing 0.05% Tween 20) for 1 hour at 22°C. Membranes were probed with primary antibodies (against LIMK, p-LIMK, cofilin, or p-cofilin, all Santa Cruz Biotechnology) diluted 1∶1000 into blocking buffer for 1 hour at 22°C. After three washes with TBS-T, membranes were incubated with anti-mouse HRP (horseradish peroxidase)-conjugated secondary antibody (GE Healthcare) diluted 1∶5000 into blocking buffer for 1 hour at 22°C. Membranes were washed three more times with TBS-T and proteins were detected using the ECL plus detection system (GE Healthcare) and a Gel Doc XR imager. Bio Rad Quantity One software was used for densitometry.

### G-actin/F-actin In Vivo Assay

Ratios of globular (G-actin) to filamentous (F-actin) in cultured, serum-starved cells were determined using the G-actin/F-actin In Vivo Assay Kit (Cytoskeleton Inc.) as described in the manufacturer's protocol. Serum-starved, untreated cells (negative control) and cells treated with F-actin enhancing solution (positive control) were analyzed alongside experimental samples (MAM-treated and controls, as described in the figure legends). G-actin and F-actin levels were determined by Western Blotting and were quantified by densitometry. Results shown are means ± s.e.m. from two independent experiments.

### GTPase activation assays

Following infection or incubation with beads, cells were washed and collected by scraping into GTPase lysis buffer (20 mM Tris HCl pH 7.5, 10 mM MgCl_2_, 150 mM NaCl, 1% Triton X-100. Lysates were homogenized and cleared by centrifugation (13000 rpm, 20 min). 500 µg of cleared lysates were added to 30 µg of GST-PAK PBD bound to glutathione agarose beads and incubated for 1 hour at 4°C. Samples were separated by SDS-PAGE and immunoblotted with α-Cdc42 or α-Rac antibodies (Sigma) and compared to total GTPase levels detected in cell lysates. Activated RhoA was pulled down with the use of a RhoA activation kit (Cytoskeleton) according to the manufacturer's instructions. Total and GTP-bound RhoA was detected following SDS-PAGE separation and Western Blotting using α-RhoA antibody (Sigma).

### Inhibition of Rho GTPase activity

To study cellular phenotypes independent of GTPase activation, cells were treated with either *Clostridium difficile* toxin B (TcdB) or C3 transferase to irreversibly inactivate either RhoA, Rac and Cdc42 or RhoA, respectively. Cells were treated wither with 200 ng/ml TcdB (List Biologicals) or 1 µg/ml cell-permeable C3 (Cytoskeleton) for 4 hours. Attachment experiments were carried out immediately after toxin treatment.
